# Correlation between hyperlipidemia and serum vitamin D levels in an adult Chinese cohort

**DOI:** 10.3389/fnut.2024.1302260

**Published:** 2024-10-21

**Authors:** Jinxiu Wang, Tala Shi, Lanlan Xu, Yanuo Li, Wei Mi, Chunyang Wang, Peng Lu, Lingyun Li, Ziyue Liu, Zhiyong Hu

**Affiliations:** ^1^School of Public Health, Binzhou Medical University, Yantai, Shandong, China; ^2^School of Basic Medicine, Binzhou Medical University, Yantai, Shandong, China; ^3^Juxian People’s Hospital, Rizhao, Shandong, China

**Keywords:** vitamin D, hyperlipidemia, total cholesterol, triglycerides, lipid metabolism

## Abstract

Vitamin D deficiency has emerged as a significant concern in public health due to its potential association with various metabolic disorders. This study aimed to investigate the relationship between serum vitamin D levels and the susceptibility to hyperlipidemia among adults. Using a multi-stage sampling approach, we recruited a cohort of 2072 eligible individuals aged over 18 years. Serum 25-hydroxyvitamin D [25(OH)D] levels were measured alongside glucolipid metabolic markers, and comprehensive demographic and physical data were collected. The cohort exhibited a hyperlipidemia prevalence of 42.18%, with 19.88% demonstrating vitamin D deficiency. Notably, 23.68% of individuals with vitamin D deficiency also presented hyperlipidemia. Statistical analysis revealed a significantly higher prevalence of hyperlipidemia among those with vitamin D deficiency compared to those with sufficient levels (23.68% vs. 17.11%, *P* < 0.05). After adjusting for various factors including age, geographical region, exercise status, BMI, fasting glucose level, and blood pressure, lower serum 25(OH)D concentrations were found to significantly increase the risk of hyperlipidemia (Odds Ratio [OR] = 1.41; 95% CI: 1.057, 1.885; *P* < 0.05). Further stratification of the hyperlipidemic cohort revealed that vitamin D deficiency was associated with 1.459- and 1.578-times higher risks for total cholesterol and triglyceride abnormalities, respectively, compared to those with sufficient vitamin D levels. Moreover, each 10 ng/mL decrease in serum vitamin D level corresponded to an increased risk of total cholesterol (OR = 0.82; 95% CI: 0.728, 0.974; *P* < 0.05) and triglyceride abnormalities (OR = 0.79; 95% CI: 0.678, 0.927; *P* < 0.05). However, there were no significant differences observed between vitamin D-sufficient and−deficient groups regarding Low-Density Lipoprotein (LDL) and High-Density Lipoprotein (HDL) abnormalities. These findings underscore the potential role of serum vitamin D deficiency as an independent risk factor contributing to the elevated prevalence of hyperlipidemia in the adult population.

## Introduction

Hyperlipidemia (HL), also known as dyslipidemia, is a common metabolic disorder characterized by abnormalities in lipids, including low-density lipoprotein cholesterol (LDL-C), high-density lipoprotein cholesterol (HDL-C), triglycerides (TG) and cholesterol (CHL). HL frequently co-occurs with obesity and type 2 diabetes (T2D) (1). Recent surveys such as the China Health and Nutrition Survey (2001–2002, 2011), the China Chronic Kidney Disease Task Force Survey (2010), and the China Population Survey on Nutrition and Chronic Diseases (2012) report HL prevalence rates among Chinese adults aged ≥ 18 years ranging from 18.6 to 40.4% (2). HL significantly diminishes quality of life and amplifies cardiovascular disease risk by two to three times (2), positioning it as a crucial target for global preventive strategies.

Vitamin D functions as a soluble steroid hormone synthesized from 7-dehydrocholesterol upon exposure to ultraviolet B (UVB) radiation in the skin, and it can also be obtained from dietary sources. Beyond its established role in calcium and phosphorus metabolism (3–5), vitamin D’s non-classical effects have gained attention, particularly its impact on lipid metabolism through identified receptors on pancreatic β cells and adipocytes (6–11). Vitamin D deficiency poses a global public health concern, correlating with obesity and related metabolic disruptions (12–14). Furthermore, studies have revealed a higher prevalence of vitamin D deficiency in individuals with T2D and HL compared to the general population (15–17), reinforcing its significant role in metabolic health.

Despite these associations, conflicting outcomes from large-scale trials on vitamin D supplementation challenge its purported benefits for conditions like hypertension, insulin sensitivity, and lipid profiles Silvia Savastano’s research has also underscored the nutritional link between lower vitamin D levels and obesity (18). However, several large-scale randomized trials examining vitamin D supplementation have reported negative outcomes (19–21), casting doubt on the potential benefits of vitamin D supplementation for hypertension (22), insulin sensitivity (23), and disturbed lipid parameters (24). This is in contrast to observational evidence, which included the physiological role of vitamin D in lipid metabolism, the findings of in vivo experimental studies, and the outcomes of clinically relevant research (12–18). Collectively, the literature indicates that vitamin D not only influences calcium and phosphorus metabolism but is also closely associated with blood lipid levels. However, the conflicting findings across studies highlight the need for more larger-scale comprehensive investigations.

This study aims to investigate the relationship between serum vitamin D levels and blood lipids among adults in southern Zhejiang, China, an area with a subtropical monsoon climate distinct from northern regions. While previous research predominantly focused on specific populations, potentially introducing selection bias, our community-based approach seeks to validate these associations more broadly. This study’s insights into vitamin D’s role in lipid regulation could inform future strategies for vitamin D supplementation in managing HL, contributing to novel preventive and therapeutic approaches within cardiovascular health.

## Objects and method

### Research objects

A multi-stage sampling method was employed for sample recruitment. Two communities/administrative villages were selected from 16 streets/towns in the Lishui area of southeastern Zhejiang Province using random integer sampling. Then, the corresponding number of participants was randomly selected from each community. The specific sampling approach was as follows. In the first step, cluster sampling was employed, with all communities/administrative villages within each street/township being assigned numerical labels. Two communities/administrative villages were selected through a random drawing process. In the second step, systematic sampling was utilized to select survey participants from the chosen communities/administrative villages. This involved sorting households by house number and using a random number generator to select the first household. Subsequent sample households were determined at intervals based on the total number of households in the community and the desired sample size. All household members over 18 years of age in the sampled households were selected as survey subjects. The study exclusion criteria included: a history of psychiatric disorders; those in a hyperosmolar coma; those using corticosteroids; patients receiving osteocalcin or vitamin D supplements; Patients with dyslipidemia who are receiving medication; cancer, chronic liver disease, chronic kidney disease, diabetes and other serious diseases; and those who were bedridden for long periods. A final sample of 2072 eligible subjects participated in this study. Study subjects provided written informed consent and the study was performed in strict adherence to the Declaration of Helsinki.

### Methods

#### Information collection

A standardized questionnaire was employed to collect demographic information from the participants, including age, gender, residential area, socioeconomic status (educational level), smoking habits (current, former, or never smoked), alcohol consumption (current, former, or never drank), physical activity, and family history of diabetes. Educational attainment was categorized into four groups: no formal education, elementary school, junior high school, and college or higher. The guidelines for physical activity set forth by the World Health Organization (a minimum of 150 min of moderate activity per week or an equivalent level of exercise) were utilized as a reference in this study.

#### Body measurements

Anthropometric measurements were conducted, including height, weight, waist circumference, hip circumference, and blood pressure. All measurements were obtained with the participants standing barefoot. Standardized procedures were employed for height and weight measurement. Weight was measured while the participants wore lightweight clothing, with a precision of 0.1 kg. Height measurement was conducted after the removal of the shoes, with a precision of 0.1 cm. The waist and hip circumference were assessed using a tape measure, accurate to 0.1 cm. Blood pressure readings were taken while participants remained seated in a quiet position for at least 15 min. A digital sphygmomanometer (HEM-7211, Omron, Japan) was utilized to obtain two consecutive blood pressure measurements, and the average was recorded as the patient’s blood pressure. The heart rate was determined by gently palpating the radial artery at the wrist using the index, middle and ring fingers of the right hand, and then counting the pulse for 30 s. This was recorded as the pulse rate.

The formula for the calculation of the body mass index (BMI) was as follows: BMI = weight (kg)/height (m)^2^. The formula for the calculation of the waist-to-hip ratio (WHR) was as follows: WHR = waist/hip circumference.

#### Detection of biochemical indices

After a 12-h fasting period, 3 ml peripheral venous blood samples were collected and centrifuged at 3000 r/min for 10 min. The upper serum layer was extracted for analysis of the relevant parameters. Serum 25(OH)D levels were determined using an electrochemiluminescence assay (Roche, COBAS e601, COBAS e601, Roche Diagnostics GMBH, Mannheim, Germany). Fasting blood glucose (FBG) levels were measured using the hexokinase method (COBAS c702, Roche Diagnostics GMBH, Mannheim, Germany), the coefficient of inter-/intra-CVs was less than 10%. Total cholesterol (TC), TG, LDL-C, and HDL-C were quantified using enzymatic assays (Roche, COBAS e601).

#### Determination of serum vitamin D level

Serum vitamin D levels were classified according to the clinical practice guidelines of the Endocrine Society as follows (11): Deficient: 25(OH)D < 20 ng/mL; Insufficient: 20 ≤ 25(OH)D < 30 ng/mL; Sufficient: 25(OH)D ≥ 30 ng/mL.

#### Determination of HL

According to the Chinese Adult Hyperlipidemia Prevention and Treatment Guidelines tailored for the Chinese population, the classification of HL was performed as follows: the patient met one or more of the following conditions: 1) TC ≥ 6.22 mmol/L; 2) TG ≥ 2.26 mmol/L; 3) HDL-C < 1.04 mmol/L; 4) LDL-C ≥ 4.14 mmol/L; 5) previously diagnosed with HL by a medical institution at the county level or above.

#### Quality control

To obtain high-quality survey data, a pre-survey was performed, and the community physicians and nursing staff who conducted the research were provided with uniform on-site training and were required to pass an assessment before they could start collecting data. After the implementation of on-site quality control, the quality of the survey data, physical measurements, blood sample collection, laboratory testing, and other aspects of the methodology were dynamically monitored in real-time. If any quality problems were discovered, timely feedback and correction were provided to prevent the further collection of biased data. Unified data preparation and entry procedures were implemented at each survey monitoring point. Two-person data entry, data logic, and abnormal data checking procedures were utilized. The duration of our study is 2 months.

#### Statistical analysis

The data were entered into EpiData 3.1 software. Dual data entry was conducted to ensure logical consistency and accuracy. Normally distributed data are presented as the mean ± standard deviation (x ± s), while non-normally distributed data are expressed as the median and interquartile range. Data were presented as mean ± standard deviation or median for continuous variables and as *n* (%) for categorical variables. Participants were divided into three groups using the serum 25(OH)D levels: Deficiency group (25(OH)D < 20 ng/mL), Insufficient group (20 ≤ 25(OH)D < 30 ng/mL) and Sufficient group (25(OH)D ≥ 30 ng/mL). The differences in quantitative variables between the two groups were compared by using independent Student’s t-tests or Wilcoxon test as appropriate. Logistic regression analysis was performed to calculate corresponding odds ratios (ORs) and adjust for multiple factors. Model 1: unadjusted; model 2: adjusted for age and gender; model 3: adjusted for model 2 and smoking, alcohol consumption, exercise, daily oil and salt intake; model 4: adjusted for model 3 and BMI, FBG, SBP and DBP. Statistical analysis was carried out using SPSS version 19.0 (SPSS, Chicago, IL, USA), with a two-sided *P*-value < 0.05 for all statistical tests.

## Results

### Sample characteristics

A total of 2072 participants who met the inclusion criteria and agreed to participate in the study were included in the study. The prevalence of HL in the sample was 42.18%. The rates of vitamin D deficiency and insufficiency were 19.88% and 57.63%, respectively. An sufficient vitamin D level was observed in only 22.49% of the participants. The prevalence of hyperlipidemia was significantly higher among those with vitamin D deficiency compared to those with sufficient vitamin D levels (50.24% vs. 37.98%, *P* < 0.05). Upon stratifying the participants according to their serum 25(OH)D levels, no differences were observed between the vitamin D-deficient, vitamin D-insufficient, and vitamin D-sufficient groups in terms of age, daily oil consumption, and daily salt intake. However, statistically significant differences were found in terms of gender, region, education level, smoking and alcohol consumption (*P* < 0.05). Regarding BMI, TC, TG, LDL-C, FBG, HL prevalence, and hyperglycemia prevalence, the vitamin D-deficient group exhibited significantly higher values than the vitamin D-sufficient group (*P* < 0.05). Refer to Table 1 for details.

**TABLE 1:** Grouping characteristics of vitamin D in the study population (*n* = 2072).

Variable	Total	Serum 25(OH)D level (ng/mL)			*F/x* ^2^	*P*-value
	(*n* = 2072)	<20 (*n* = 412)	20–29 (*n* = 1194)	≥ 30 (*n* = 466)		
25(OH)D level	25.26 ± 6.48	16.16 ± 2.93	25.08 ± 2.76	33.76 ± 3.75		
**1. Basic index**						
Age (years)	52.65 ± 13.89	52.28 ± 15.29	52.09 ± 13.64	53.56 ± 13.19	2.406	0.090
Gender (Male), n (%)	887 (42.81)	108 (26.21)	482 (40.37)	297 (63.73)	132.59	< 0.001
Region (urban area), n (%)	1278 (61.68)	287 (69.66)	741 (62.06)	250 (53.65)	23.893	< 0.001
Education (college or above), n(%)	148 (7.14)	37 (8.98)	93 (7.79)	18 (3.86)	29.54	0.003
Average daily intake of oil	52.19 ± 30.45	50.54 ± 29.73	52.36 ± 30.33	53.25 ± 31.36	0.908	0.403
Average daily salt intake	11.81 ± 6.73	11.99 ± 6.88	11.88 ± 6.67	11.50 ± 6.74	0.708	0.493
Current smoker, n(%)	448 (21.64)	71 (17.32)	237 (19.85)	140 (30.04)	32.734	< 0.001
Current drinkers, n(%)	628 (30.37)	86 (20.92)	356 (29.82)	186 (40.17)	42.827	< 0.001
**2. Metabolic index**						
Body mass index (kg/m^2^)	23.83 ± 3.36	23.91 ± 3.46*	24.02 ± 3.42*	23.27 ± 3.06	8.485	0.001
< 24	1151 (55.55)	220 (53.39)	626 (52.43)	305 (65.45)	23.983	<0.001
≥ 24	921 (44.45)	192 (46.60)	568 (47.57)	161 (34.55)		
Total cholesterol	5.44 ± 1.08	5.75 ± 1.24*	5.41 ± 1.03*	5.26 ± 1.02	24.600	< 0.001
triglyceride	2.09 ± 0.90	2.51 ± 2.92*	2.09 ± 3.31*	1.74 ± 1.25	7.923	< 0.001
Low-density lipoprotein	3.18 ± 0.84	3.33 ± 0.89*	3.17 ± 0.82*	3.05 ± 0.84	11.932	< 0.001
High-density lipoprotein	1.52 ± 0.39	1.56 ± 0.45 *	1.51 ± 0.36*	1.53 ± 0.38	3.280	0.038
Dyslipidemia, *n* (%)	874 (42.18)	207 (50.24) *	490 (41.04) *	177 (37.98)	14.986	0.001
Fasting blood glucose (mmol/L)	5.93 ± 1.60	6.14 ± 2.42*	5.92 ± 1.32	5.76 ± 1.29	5.948	0.003
Hyperlipidemia *n* (%)	137 (6.61)	39 (9.47) *	75 (6.28) *	23 (4.94)	7.767	0.021
Systolic blood pressure	126.97 ± 18.65	127.44 ± 18.73	126.39 ± 18.53	128.04 ± 18.84	1.468	0.231
Diastolic blood pressure	79.71 ± 10.43	79.16 ± 10.52	80.05 ± 10.55	79.35 ± 10.01	1.489	0.226
Hypertension, *n* (%)	241 (11.63)	43 (10.44)	149 (12.48)	49 (10.52)	1.972	0.373

*Compared with 25(OH)D adequate group (serum 25(OH)D ≥ 30 ng/ml), the difference was significant; In analysis of variance (ANOVA), an F value greater than 1 indicates significant differences between groups. Similarly, in the chi-square test, a larger χ^2^ value implies a more significant difference between observed and expected frequencies.

### Characteristics of the HL subgroups

The prevalence of serum 25(OH)D deficiency was significantly higher among individuals with HL compared to those with normal blood lipid levels (23.68% vs. 17.11% ng/mL, *P* < 0.05). There were no significant differences between the two groups in terms of gender, education level, daily oil consumption, daily salt intake, smoking and alcohol consumption. However, statistically significant differences were observed between the two groups in terms of age, region and exercise habits. In terms of metabolic indicators such as BMI, FBG, systolic blood pressure (SBP), diastolic blood pressure (DBP), hyperglycemia prevalence, and hypertension prevalence, the vitamin D-deficient group exhibited significantly higher values than the vitamin D-sufficient group (*P* < 0.05). Refer to Table 2 for details.

**TABLE 2:** Characteristics of hyperlipidemia subgroups in the study population (*n* = 2072)^a^.

	Hyperlipidemia		*t/z/x* ^2^	*P*-value
	Yes (874)	No (1198)		
25(OH)D deficiency (yes)	207 (23.68 %)	205 (17.11 %)		
25(OH) Insufficient D (yes)	490 (56.07 %)	704 (58.77 %) 289 (24.12 %)	14.986	0.001
25(OH)D sufficient (yes)	177 (20.25 %)			
**1. Basic index**				
Age (years)	53.86 ± 13.35	51.78 ± 14.22	−3.369	0.001
Gender (Male), n(%)	362 (41.42 %)	525 (43.82 %)	1.193	0.275
Region (urban area), n(%)	598 (68.42 %)	680 (56.76 %)	29.066	<0.001
Education (college or above), n(%)	42 (4.80 %)	58 (4.84 %)	2.769	0.837
Average daily intake of oil	51.71 ± 30.85	52.55 ± 30.15	0.619	0.536
Average daily salt intake	11.91 ± 6.98	11.75 ± 6.54	−0.534	0.593
Current smoker, n(%)	181 (20.73%)	267 (29.82%)	2.927	*0.231*
Current drinkers, n(%)	260 (29.82%)	368 (30.77%)	1.339	0.512
Exercise regularly, n(%)	288 (32.95%)	288 (24.08%)	19.79	<0.001
**2. Metabolic index**				
Body mass index (kg/m^2^)	24.58±3.39	23.28±3.34	−8.884	<0.001
<24	396 (66.10%)	755 (69.73%)	64.209	<0.001
=24	478 (45.31%)	443 (36.98%)		
Fasting blood glucose (mmol/L)	6.14±2.05	5.78±1.15	−4.684	<0.001
Hyperglycemia, n(%)	78 (8.92%)	59 (5.18%)	13.092	<0.001
SBP (mmHg)	129.58±18.14	125.08±18.79	−5.462	<0.001
DBP (mmHg)	80.93±10.57	78.83±10.24	−4.572	<0.001
Hypertension, *n* (%)	115 (13.16%)	126 (10.52%)	3.428	*0.064*

The italicized values represent the statistical tests used for group comparisons. For normally distributed data, the *t*-test was applied for independent sample comparisons. For non-normally distributed data, the *Z*-test was used. When data are expressed as frequencies *(n)* and percentages (%), the χ2 test was employed for comparisons between groups.

^a^Data are mean±SD, n (%), or median (interquartile range).

^b^BMI, body mass index; SBP, Systolic blood pressure; DBP, Diastolic blood pressure; FPG, Fasting plasma glucose; In all cases, significance was considered at *p* < 0.05.

### Multifactorial analysis of the correlation between 25(OH)D levels and HL

Comparatively, the vitamin D-deficient group had a 1.65-fold higher risk of developing HL compared to the vitamin D-sufficient group. Additionally, the OR for every 10 ng/mL decrease in serum 25(OH)D levels was 0.78, with a statistically significant *P*-value. This indicates that for every 10 ng/mL reduction in serum 25(OH)D levels, the risk of developing HL increased by 78%, meaning the risk was 1.78 times higher compared to baseline (*P* < 0.05, as illustrated in Table 3). These findings retained statistical significance even after adjusting for multiple confounding factors. Following adjustments for age, gender, residence, and education, the deficient group exhibited a 1.55-fold higher risk of HL compared to the sufficient group. Subsequent adjustments for smoking, alcohol consumption, exercise, daily oil and salt intake showed a 1.54-fold higher risk for the deficient group. Further adjustment for BMI, FBG, SBP, and DBP sustained a 1.41 times higher risk in the deficient group. Additionally, the OR for every 10 ng/mL decrease in serum 25(OH)D levels was 0.84, indicating that the risk of developing HL increased by 84%. Thus, the risk of HL was 1.84 times higher compared to baseline (*P* < 0.05, as displayed in Table 3). The incidence of TC in vitamin D sufficient group and vitamin D deficiency group was 18.88% and 28.88%, respectively. Compared to the vitamin D-sufficient group, the deficient group exhibited a 1.75-fold higher risk of developing TC; Additionally, The OR for every 10 ng/mL decrease in serum 25(OH)D levels was 0.75, indicating that the risk of total TC increased by 75%, and the risk of TC was 1.75 times higher compared to baseline. Further adjustments for smoking, alcohol consumption, exercise, daily oil and salt intake revealed a 1.51-fold higher risk for the deficient group. After further adjustment for BMI, FBG, SBP and DBP, the risk remained 1.46 times higher in the deficient group. Additionally, the OR for every 10 ng/mL decrease in serum 25(OH)D levels was 0.82, indicating that the risk of total TC increased by 82%, and the risk of TC was 1.82 times higher than baseline (*P* < 0.05, as shown in Table 4).

**TABLE 3:** Multivariate corrected ratios (ORs) of serum 25-hydroxyvitamin D levels and 95% for hyperlipidemia (*n* = 2072).

	Serum 25(OH)D levels		*P* for linear trend	Change in ORs and 95% CI for hyperlipidemia per 10 ng/mL decrease in serum 25(OH)D concentrations
	Sufficient (≥ 30 ng/mL)	Lack of (20 ng/mL)		
Mean value (ng/ml)	33.77 ± 3.75	16.16 ± 2.93		
**Hyperlipidemia**				
Unadjusted prevalence rate (%) (%)	37.98 %	50.24 %	< 0.001	
Model 1	1.00	1.649 (1.260, 2.157)	< 0.001	0.780 (0.682, 0.893)
Model 2	1.00	1.547 (1.169, 2.048)	0.002	0.805 (0.699, 0.927)
Model 3	1.00	1.536 (1.157, 2.039)	0.003	0.808 (0.700, 0.931)
Model 4	1.00	1.412 (1.057, 1.885)	0.020	0.842 (0.728, 0.974)

Model 1: unadjusted; model 2: adjusted for age and gender; model 3: adjusted for model 2 and smoking, alcohol consumption, exercise, daily oil and salt intake; model 4: adjusted for model 3 and BMI, FBG, SBP and DBP. OR, odds ratio; CI, confidence interval.

**TABLE 4:** Multivariate corrected ratios (ORs) of serum 25-hydroxyvitamin D levels and 95% for TC, TG, LDL and HDL (*n* = 2072).

	Serum 25(OH)D levels		*P for* *linear trend*	Change in OR and 95% CI for hyperlipidemia per 10 ng/mL decrease in serum 25(OH)D concentration
	sufficient (≥ 30 ng/mL)	Lack of (20 ng/mL)		
**1. Abnormal total cholesterol**				
Unadjusted prevalence rate (%) (%)	18.88 %	28.88 %	< 0.001	
Model 1	1.00	1.745 (1.273, 2.390)	0.001	0.750 (0.638, 0.882)
Model 2	1.00	1.521 (1.095, 2.115)	0.013	0.802 (0.678, 0.949)
Model 3	1.00	1.505 (1.078, 2.101)	0.016	0.805 (0.679, 0.955)
Model 4	1.00	1.459 (1.043, 2.040)	0.027	0.817 (0.688, 0.970)
**2. Abnormal triglyceride**				
Unadjusted prevalence rate (%) (%)	22.75 %	33.50%	< 0.001	
Model 1	1.00	1.711 (1.270, 2.304)	< 0.001	0.763 (0.657, 0.887)
Model 2	1.00	1.784 (1.308, 2.434)	< 0.001	0.747 (0.640, 0.873)
Model 3	1.00	1.774 (1.296, 2.429)	< 0.001	0.750 (0.640, 0.877)
Model 4	1.00	1.578 (1.144, 2.177)	0.005	0.793 (0.674, 0.932)
**3. Abnormal low density lipoprotein**				
Unadjusted prevalence rate (%) (%)	10.52 %	15.05 %	0.099	
Model 1	1.00	1.508 (1.010, 2.250)	0.045	0.810 (0.661, 0.994)
Model 2	1.00	1.246 (0.821, 1.891)	0.301	0.891 (0.721,1.101)
Model 3	1.00	1.192 (0.784, 1.814)	0.412	0.910 (0.735,1.126)
Model 4	1.00	1.123 (0.736, 1.713)	0.591	0.936 (0.754,1.161)
**4. High density lipoprotein abnormality**				
Unadjusted prevalence rate (%) (%)	6.65 %	8.01 %	0.720	
Model 1	1.00	1.222 (0.734, 2.033)	0.441	0.905 (0.701, 1.167)
Model 2	1.00	1.375 (0.810, 2.334)	0.238	0.853 (0.655, 1.111)
Model 3	1.00	1.328 (0.780, 2.263)	0.296	0.868 (0.666, 1.132)
Model 4	1.00	1.176 (0.685, 2.019)	0.556	0.922 (0.703, 1.208)

Model 1: unadjusted; model 2: adjusted for age and gender; model 3: adjusted for model 2 and smoking, alcohol consumption, exercise, daily oil and salt intake; model 4: adjusted for model 3 and BMI, FBG, SBP and DBP. OR, odds ratio; CI, confidence interval.

The incidence of TG in vitamin D sufficient group and vitamin D deficiency group was 22.75% and 33.50%, respectively. Compared to the vitamin D-sufficient group, the deficient group exhibited a 1.71-fold higher risk of developing TG. Additionally, the OR for every 10 ng/mL decrease in serum 25(OH)D levels was 0.76, indicating that the risk of total TG increased by 76%, and the risk of TG was 1.76 times higher than before (*P* < 0.05, as shown in Table 4). Further adjustments for smoking, alcohol consumption, exercise, daily oil and salt intake revealed a 1.77-fold higher risk for the deficient group. After further adjustment for BMI, FBG, SBP and DBP, the risk remained 1.58 times higher in the deficient group. Additionally, the OR for every 10 ng/mL decrease in serum 25(OH)D levels was 0.79, indicating that the risk of total TG increased by 79%, and the risk of TG was 1.79 times higher than before (*P* < 0.05, as shown in Table 4).

Furthermore, The incidence of LDL-C in vitamin D sufficient group and vitamin D deficiency group was 10.52% and 15.05%, respectively. The incidence of HDL-C in vitamin D sufficient group and vitamin D deficiency group was 6.65% and 8.01%, respectively. However, no significant differences between the vitamin D-sufficient and deficient groups were observed in the stratified analysis of LDL-C and HDL-C (*P* trend > 0.05, as shown in Table 4).

## Discussion

This study found that the prevalence of hyperlipidemia was significantly higher in people with vitamin D deficiency than in people with sufficient vitamin D levels, and lower serum 25(OH)D concentration significantly increased the risk of hyperlipidemia, especially the risk of total cholesterol and triglyceride abnormalities. The current study identified a compelling association between diminished serum 25(OH)D concentrations and elevated susceptibility to HL. This supports the notion that vitamin D plays an intricate role in the regulation of lipid metabolism, thus having an influential impact on the pathogenesis of HL. This finding is consistent with the findings of similar studies. For example, Joshua F. Baker (25) revealed a robust correlation between vitamin D deficiency and the emergence of HL. Similarly, a prospective cohort study of 6740 participants in the United States aged 20–80 years demonstrated that serum 25(OH)D levels were positively correlated with heightened HDL-C and TC levels but inversely correlated with TG and LDL-C concentrations (26). Similarly, an exploratory study of elderly Koreans’ health status (27) revealed that lower vitamin D levels (ranging from 14.20 to 18.99 ng/mL in men and 11.20 to 15.59 ng/mL in women) were associated with increased waist circumference, hypertriglyceridemia, and elevated LDL-C. This finding remained after meticulous adjustment for potential confounders, including age, residential region, season, and pertinent habits such as exercise, smoking, and alcohol consumption. Moreover, Zhu and Heil’s (28) study of Shanghai residents aged 19–70 years revealed that 50% of the study participants exhibited suboptimal 25(OH)D levels. Moreover, a linear inverse correlation was observed between serum 25(OH)D concentrations and lipid concentrations. Specifically, for each 1 ng/mL increment in the 25(OH)D level, there were significant reductions in the TC and LDL-C levels. Similarly, Barbalho et al. (29) reported that 80% of patients within a cardiology department had vitamin D deficiency. Moreover, these deficient patients exhibited elevated glucose, glycosylated hemoglobin, TC, LDL-C, TG, atherosclerotic indices, and BMI, as compared to those with sufficient vitamin D levels. Taken together, this body of literature supports the vital role of vitamin D in the regulation of lipid metabolism. These findings also highlight the broader clinical ramifications of vitamin D inadequacy in diverse medical contexts. Vitamin D inadequacy has been implicated in conditions such as asthma (30), COVID-19 infection (31) and hyperuricemia (32). In these conditions, vitamin D supplementation has been linked with improved clinical outcomes. Thus, there is an emerging consensus that vitamin D might not influence the development of these diseases per se, but rather, a deficiency in vitamin D may have a significant influence on the quality of life of patients through various pathways.

Numerous complex mechanisms underlie the intricate relationship between vitamin D and HL. Studies have found that vitamin D can be stored in fat cells, and vitamin D may promote energy consumption and reduce lipid levels by increasing fatty acid oxidation and mitochondrial metabolism (33, 34). Studies have also shown that vitamin D plays an important role in adiponectin secretion. adiponectin (APN) is the only protein product found so far that is secreted by adipocytes in response to the increase of fat volume, and the whole genome scan shows that the adiponectin gene fragment region has a susceptibility site for metabolic syndrome (35). Adiponectin activates downstream AMP-activated protein kinase (AMPK) and PPARα-mediated signal transduction pathways by binding to specific receptors, namely Adipo R1 and Adipo R2. Thus, it plays various biological roles such as regulating glucose and lipid metabolism, protecting vascular endothelium and anti-inflammation (35, 36). Vitamin D receptors on adipocytes show a direct mechanism of action of vitamin D in adiponectin gene expression, and vitamin D may increase serum adiponectin levels by decreasing TNF-α gene expression (36). At the same time, vitamin D, as a negative regulator of renin expression and renin-angiotensin system activity, may promote adiponectin production by reducing angiotensin production (35). The study by Chow et al. (37) indicated that the vitamin D receptor (VDR) is a novel target for cholesterol modulation interventions. Following 1,25(OH)2D3 treatment, this study observed an upsurge in cholesterol 7α-hydroxylase (CYP7A1) expression. The prevailing modulatory feedback loop for CYP7A1 centers on the bile acid receptor (FXR, NR1H4) and the small heterodimeric chaperone (SHP, NR0B2). FXR activation, triggered by bile acids like chenodeoxycholic acid (CDCA), promotes SHP expression, and this atypical nuclear receptor orchestrates a cascade that curbs CYP7A1 via the inhibition of viral transcription factors, namely, liver receptor homolog-1 (LRH-1, NR5A2) and hepatocyte nuclear factor 4α (HNF-4α, NR2A1) (38). A compelling body of evidence also highlights the role of the Fxr-independent, Shp-dependent paradigm, wherein Vdr-mediated down-regulation of Shp amplifies Cyp7a1 expression, thereby inducing cholesterol reduction. Moreover, a constellation of in vitro investigations across diverse species—mice, pigs, chickens and humans—has revealed that vitamin D has an inhibitory influence on the adipocyte differentiation process, consequently reducing adipogenesis (39). The key adipogenic orchestrator, peroxisome proliferator-activated receptor γ (PPARγ), exhibits shared binding partners with VDR, in particular, the retinoid X-like receptor (RXR), thereby generating a competitive dynamic between PPARγ and RXR (40). Enhanced VDR expression reduces the window for adipogenesis-related gene expression, inherently limiting the proliferative expansion of adipocyte clones. In porcine-derived preadipocytes, 1,25(OH)2D3 not only impedes cell differentiation but also suppresses the mRNA expression of PPARγ and pivotal adipocyte marker genes, namely, Lpl, Pck2, Scd and Glut4 (41). While the inhibitory effect of 1,25(OH)2D3 on preadipocyte differentiation likely contributes to its antilipidemic effect, it may potentially operate through the wingless MMTV integration site/β-catenin pathway. This pathway, pivotal to an array of cellular functions, has emerged as an inhibitor of adipogenesis (42, 43). Furthermore, the intricate interplay between vitamin D and lipid metabolism is consistent with its stimulation of insulin receptor expression (44) and the documented association between low vitamin D levels and β-cell dysfunction (45). A novel presumption of the association between vitamin D deficiency and HL may pertain to its modulation of hepatic lipid metabolism (46). Notably, vitamin D augments intestinal calcium absorption, potentially giving rise to calcium-fatty acid complexes that impede lipid absorption. Consequently, aberrant lipid processing may ensue as a consequence of vitamin D insufficiency-induced alterations in calcium availability (47). Last but not least, it is imperative to consider the profound influence of ultraviolet B (UVB) radiation-driven cutaneous cholecalciferol synthesis—primarily via sunlight exposure—as the principal source of vitamin D (80–90%) (14). Pertinently, the observed reduced 25(OH)D concentrations in obese individuals—often measured in experimental contexts due to its extended half-life—can be attributed to reduced sun exposure as a result of reduced mobility, limited outdoor activities, and wearing of specific high-coverage clothing items, as compared with their normal-weight counterparts (48, 49).

The role of vitamin D in lipid metabolism has been the subject of numerous studies, including several randomized controlled trials (RCTs). Some RCTs have reported positive effects of vitamin D supplementation on lipid profiles. For example, Zittermann et al. (50) demonstrated improvements in total cholesterol and LDL-C levels in overweight and obese subjects following vitamin D supplementation. Similarly, Wang et al. (51) observed beneficial effects on lipid parameters in patients with type 2 diabetes.

However, other trials have not found significant changes. Maki et al. (52) conducted a study in healthy adults and reported no significant impact of vitamin D supplementation on serum lipid levels. These mixed results suggest that the effect of vitamin D on lipid metabolism may vary depending on factors such as the study population, baseline vitamin D status, dosage, and duration of supplementation.

Despite these inconsistencies, the overall body of evidence indicates a potential role for vitamin D in regulating lipid metabolism, warranting further investigation through well-designed, large-scale RCTs.

Adipose tissue serves as a reservoir for vitamin D, leading to its sequestration and reduced bioavailability in circulation. This sequestration effect is particularly pronounced in individuals with high BMI, contributing to lower serum 25-hydroxyvitamin D [25(OH)D] levels and an increased risk of vitamin D deficiency (53). The stored vitamin D in adipose tissue may not be readily available for metabolic processes, exacerbating the deficiency observed in obese individuals (54).

Chronic inflammation, common in metabolic syndrome and diabetes, increases the body’s demand for vitamin D. Vitamin D plays a crucial role in modulating immune responses and reducing inflammation. As a result, the heightened inflammatory state in these conditions may lead to a higher consumption of vitamin D, further lowering its serum levels.

The causality between low vitamin D status and HL remains unclear and appears to be bidirectional. On one hand, vitamin D deficiency may contribute to dysregulated lipid metabolism through its effects on insulin sensitivity, pancreatic β cell function, and inflammatory pathways. This could increase the risk of developing HL. On the other hand, the low vitamin D levels observed in individuals with HL could result from increased vitamin D deposition in adipose tissue and an elevated requirement to counteract inflammation.

Epidemiological studies (55) have shown an association between low vitamin D levels and the prevalence of metabolic disorders, including HL. However, the results from randomized controlled trials (56) on vitamin D supplementation have been mixed, with some studies reporting no significant impact on lipid profiles. This discrepancy highlights the need for further research to elucidate the mechanisms underlying the relationship between vitamin D and lipid metabolism.

The findings of this study provide strong evidence for the association between vitamin D and HL, as well as between vitamin D and other pertinent lipid indices. These associations remained statistically significant even after comprehensive adjustment for potential confounding variables. Thus, these findings imply that vitamin D potentially acts as an independent risk factor for the development of HL. However, due to the limitations of observational studies, it cannot be ruled out that other confounding influenced these findings. Therefore, the causal relationship between vitamin D levels and lipid levels needs to be confirmed by further in vivo and in vitro experiments.

This study utilized a large sample obtained from a community setting. This enhances the generalizability of the findings. Nonetheless, several limitations of this research deserve consideration. Notably, there was a high prevalence of low vitamin D among the study sample, and this limited the comprehensive exploration of the relationship between vitamin D levels within the normal range and HL. Moreover, genetic factors were not accounted for in the analysis. Given the intricate links between vitamin D, obesity and HL, large-scale randomized placebo-controlled trials are necessary to corroborate the associations and evaluate the potential beneficial effects of vitamin D supplementation (57, 58). Given the diverse dietary habits and sunlight exposure across different regions worldwide, these findings may not be generalizable to populations in other countries. Thus, further extensive investigations are recommended. Additionally, since previous studies have largely focused on the clinical manifestations of diseases rather than the underlying characteristics, assessing quantitative outcomes such as anthropometric and lipid measurements within a sample predominantly composed of healthy and sub-healthy individuals offers the opportunity to broaden our understanding of the pathological and physiological pathways of obesity and HL. This insight will contribute to the advancement of global human physical health and will serve as a valuable reference for subsequent research endeavors.

## Conclusion

The results of this study suggest that the prevalence of hyperlipidemia is higher in the population with low levels of 25(OH)D, and lower serum 25(OH)D concentration significantly increases the risk of total cholesterol and triglyceride abnormalities. Serum vitamin D deficiency may be an independent risk factor for the increased prevalence of hyperlipidemia in adults. We suggest that vitamin D should be supplemented in people with vitamin D deficiency to reduce the occurrence of hyperlipidemia.

## Data Availability

The raw data supporting the conclusions of this article will be made available by the authors, without undue reservation.
